# Structured illumination microscopy imaging reveals localization of replication protein A between chromosome lateral elements during mammalian meiosis

**DOI:** 10.1038/s12276-018-0139-5

**Published:** 2018-08-28

**Authors:** Seobin Yoon, Eui-Hwan Choi, Jung-Woong Kim, Keun P. Kim

**Affiliations:** Department of Life Sciences, Chung-Ang University, Seoul, 06974 Korea

## Abstract

An important event enabling meiotic prophase I to proceed is the close juxtaposition of conjoined chromosome axes of homologs and their assembly via an array of transverse filaments and meiosis-specific axial elements into the synaptonemal complex (SC). During meiosis, recombination requires the establishment of a platform for recombinational interactions between the chromosome axes and their subsequent stabilization. This is essential for ensuring crossover recombination and proper segregation of homologous chromosomes. Thus, well-established SCs are essential for supporting these processes. The regulation of recombination intermediates on the chromosome axis/SC and dynamic positioning of double-strand breaks are not well understood. Here, using super-resolution microscopy (structured illumination microscopy), we determined the localization of the replication protein A (RPA) complex on the chromosome axes in the early phase of leptonema/zygonema and within the CEs of SC in the pachynema during meiotic prophase in mouse spermatocytes. RPA, which marks the intermediate steps of pairing and recombination, appears in large numbers and is positioned on the chromosome axes at the zygonema. In the pachynema, RPA foci are reduced but do not completely disappear; instead, they are placed between lateral elements. Our results reveal the precise structure of SC and localization dynamics of recombination intermediates on meiocyte chromosomes undergoing homolog pairing and meiotic recombination.

## Cell division: Recombination in action

Using very high resolution microscopy, Korean researchers are able to visualize meiosis, a two-step division process that ensures that each egg or sperm cell has a single set of chromosomes, in unprecedented detail. After DNA replication and before the first division cycle, pairs of chromosomes align to exchange genetic material, a process known as recombination. This process is facilitated by multi-protein structures called synaptonemal complexes (SCs) that hold the chromosomes together. Keun Kim and colleagues at Chung-Ang University, Seoul, South Korea used structured illumination microscopy to observe SCs during meiosis in mouse sperm-forming cells. They found that the position of replication protein A, a key regulator of recombination, on the SC changes as recombination takes place. These findings suggest that RPA location on SCs could be used to monitor the progression of meiotic recombination.

## Introduction

During meiosis, replicated chromosomes search for their homologous templates (also known as homologs) and form synapses to undergo meiotic recombination, a process that is essential for producing crossover (CO) and assuring correct chromosome separation during the first meiotic division^1,2^. Throughout the first meiotic prophase, sister chromatids are held together by sister chromatid cohesion, which involves the formation of a chromosome-associated multi-subunit protein complex^3–6^. Programmed double-strand breaks (DSBs), the generation of which is catalyzed by Spo11, initiate meiotic recombination and search for homologous counterpart DNA; next, the paired homologs form SCs, prominent proteinaceous structures that assemble between homologous chromosomes during meiotic prophase^2,4,7^. Meiotic prophase I is categorized into four stages defined by chromosome structures and nucleation/polymerization of the SCs: leptonema (development of axes, initiation of homolog pairing, followed by initiation of DSB); zygonema (synapsis of homologs and initiation of SC); pachynema (completion of synapsis and formation of full-length SC); and diplonema (observable chiasmata)^1,8–10^.

The SC shows a general tripartite ladder-like structure with two parallel lateral elements (LEs) where the homolog chromatin is closed and a central element (CE) that is in the gap between LEs. Ultrastructural analysis of the SC has revealed that the CE consists of transverse filaments (TFs), which are two interconnected LEs that are essential for crossing over^11–13^. The SC begins to form during the leptonema stage of meiotic prophase I as the first homologs become connected by a central region composed of TFs, which become visible between axial elements. The axial element forms a proteinaceous structure related to the two sister chromatids of the homologs. During the zygonema-to-pachynema transition, the LEs and the CEs are completely assembled in a process known as synapsis. Synapsis is fully completed at the mid-pachynema of meiosis I; subsequently, the homologs separate, and then the SCs disassemble in diplonema^3,8^.

In mammals, the core components required for forming the SC have been identified as SC protein 1 (SYCP1), SYCP2, and SYCP3. SYCP1 is involved in the TF formation of SCs. Further, SYCP2 and SYCP3, known as LE proteins, form the axial elements during leptonema^14–16^. When the homologs become synapsed during the zygonema, the axial elements are joined by the TFs composed of SYCP1^17^.

Replication protein A (RPA), a heterotrimeric protein complex consisting of the RPA1, RPA2, and RPA3 subunits, tightly binds to single-stranded DNA during replication and DNA repair in the eukaryotic cell cycle. RPA generally inhibits secondary structure of a DSB end until recombinase displaces the RPA and initiates recombination. Specifically, RPA is involved in chromosome axis-bridge formation between homologs during meiotic prophase I^7^. Thus, high-resolution cytological imaging for chromosome morphogenesis may be essential for improving the understanding of how RPA is assembled on chromosome to induce morphological changes, in addition to observing chromosome morphogenesis related to SC components and investigating the dynamics of how RPA plays crucial roles in meiotic recombination and checkpoint signaling. However, the results of studies using conventional microscopy are limited because the diffraction limits the resolution, preventing precise observation of subcellular localization and chromosome structures.

Various techniques have been developed to overcome the limitations of conventional methods. The most frequently used high-resolution techniques are structured illumination microscopy (SIM), stimulated emission depletion, and photo-activated localization microscopy; these techniques have been used to analyze molecular structures and their localization^18,19^. SIM imaging, an adapted wide-field imaging technique, uses patterned illumination to excite fluorescence samples. The emitted fluorescence signals are recorded for a range of stripe patterns (also known as illumination stripes). The superimposition between the illumination pattern and sample generates a so-called moiré pattern that gives rise to dark and light stripes in the images. The moiré pattern is created by the interaction between the high frequency of the stripe pattern and high-frequency organization of the objects within the sample containing higher-frequency information. This causes the expansion frequency space to become visible, and the resolution in two or three dimensions is improved by approximately twofold because of the reduction in the size of the point-spread function. To restructure a final image, several raw images (moiré pattern images) are collected, each of which is captured from different angles of the structured illumination^20,21^.

Using super-resolution SIM, we investigated the dynamic architecture of SCs, which are assembled between homologous chromosomes. We also specified the dynamic localization of RPA that binds to single-stranded DNA at meiotic recombination sites associated with SCs during prophase I. We describe new features of the chromosome structure and reveal the RPA focus dynamics on the chromosome axes and SC nucleation regions. Importantly, this information provides insight into how the SC structure and DSBs/single-stranded DNA (ssDNA) are regulated in mammalian meiosis.

## Materials and methods

### Mice

C57/BL6J mice were purchased from Jackson Laboratory (Bar Harbor, ME, USA) via Orient Bio (Seongnam, Korea) and were maintained under a 12-h light/dark cycle with free access to food and water. The testes of 4-week-old mice were dissected and rapidly isolated on an ice plate with phosphate-buffered saline (PBS).

### Chromosome spreads of mouse spermatocytes

We slightly modified the “drying-down technique” to prepare spermatocytes^22^. Seminiferous tubules were transferred to hypotonic buffer (30 mM Tris-HCl at pH 8.2, 17 mM sodium citrate, 5 mM ethylenediaminetetraacetic acid, 50 mM sucrose, 5 mM dithiothreitol, and 0.5 mM phenylmethylsulfonyl fluoride) and incubated at room temperature for 30 min. Swollen tubules were fixed in 4% paraformaldehyde (pH 6.9) for 5 min at room temperature. Fixed tubules were washed with PBS for 10 min. Washed tubules were then transferred to fresh PBS and disrupted via vigorous pipetting to obtain a cloudy suspension. Next, 100 mM sucrose was dropped onto a clean slide. The cell suspension was mixed with the sucrose drop on the clean slide and spread over the slide to dry more than 4 h. After drying, the slide was rinsed by immersion in 0.4% Photo-Flo (Eastman Kodak Company, Hernel Hempstead, UK, 146-4510) and distilled water. The washed slide was dried overnight at room temperature.

### Immunofluorescence microscopy analysis

The testicular cell spread slide was washed with washing buffer (0.1% Tween-20 in PBS) and then incubated in blocking buffer (1% bovine serum albumin in PBS) for 30 min at room temperature in a wet chamber. We then added a combination of primary antibodies diluted in blocking buffer and incubated the slides overnight at room temperature in a wet chamber. The primary antibodies used in this study were as follows: SYCP3 (1:500), SYCP1 (1:500) (gift from National Cancer Center in Korea), RPA (Millipore, Billerica, MA, USA, NA19L, 1:200), HORMAD1 (Proteintech, Chicago, IL, USA, 13917-1-ap, 1:200), RPA (Cell Signaling Technology, Danvers, MA, USA, #2208, 1:200), and γH2AX (Cell Signaling Technology, #2577, 1:200). After washing with washing buffer for 20 min, we incubated the slides with secondary antibodies, which were diluted in blocking buffer, for 3 h at room temperature in a wet chamber under dark conditions. The secondary antibodies used for immunofluorescence staining were as follows: Alexa-Fluor 488 (Jackson ImmunoResearch, West Grove, PA, USA, 115-545-003, 1:500), fluorescein isothiocyanate (Jackson ImmunoResearch, 112-095-003, 1:500), and tetramethylrhodamine-isothiocyanate (Jackson ImmunoResearch, 111-025-144, 1:500). After secondary antibody treatment, the slides were washed with washing buffer for 20 min. All solution remaining on the slides was dried completely, and the slides were covered with mounting solution (90% glycerol, 0.1 M Tris-HCl (pH 8.0), 0.5% *n*-propyl gallate, and 2 μg/μL 4′,6-diamidino-2-phenylindole), followed by sealing of the slide glasses with nail polish. We examined the stained slides under a fluorescence microscope (Nikon Eclipse Ti-E, Tokyo, Japan) and captured images using a microscope camera (Nikon DS-Qi2). Super-resolution images were obtained by SIM (Nikon Eclipse Ti-E) equipped with EM CCD camera iXon897 and ×100 oil objective (NA1.49). Image stacks were reconstructed by the Nikon NIS software.

## Results

### Super-resolution microscopy reveals the process of SC assembly

**Fig. 1:**
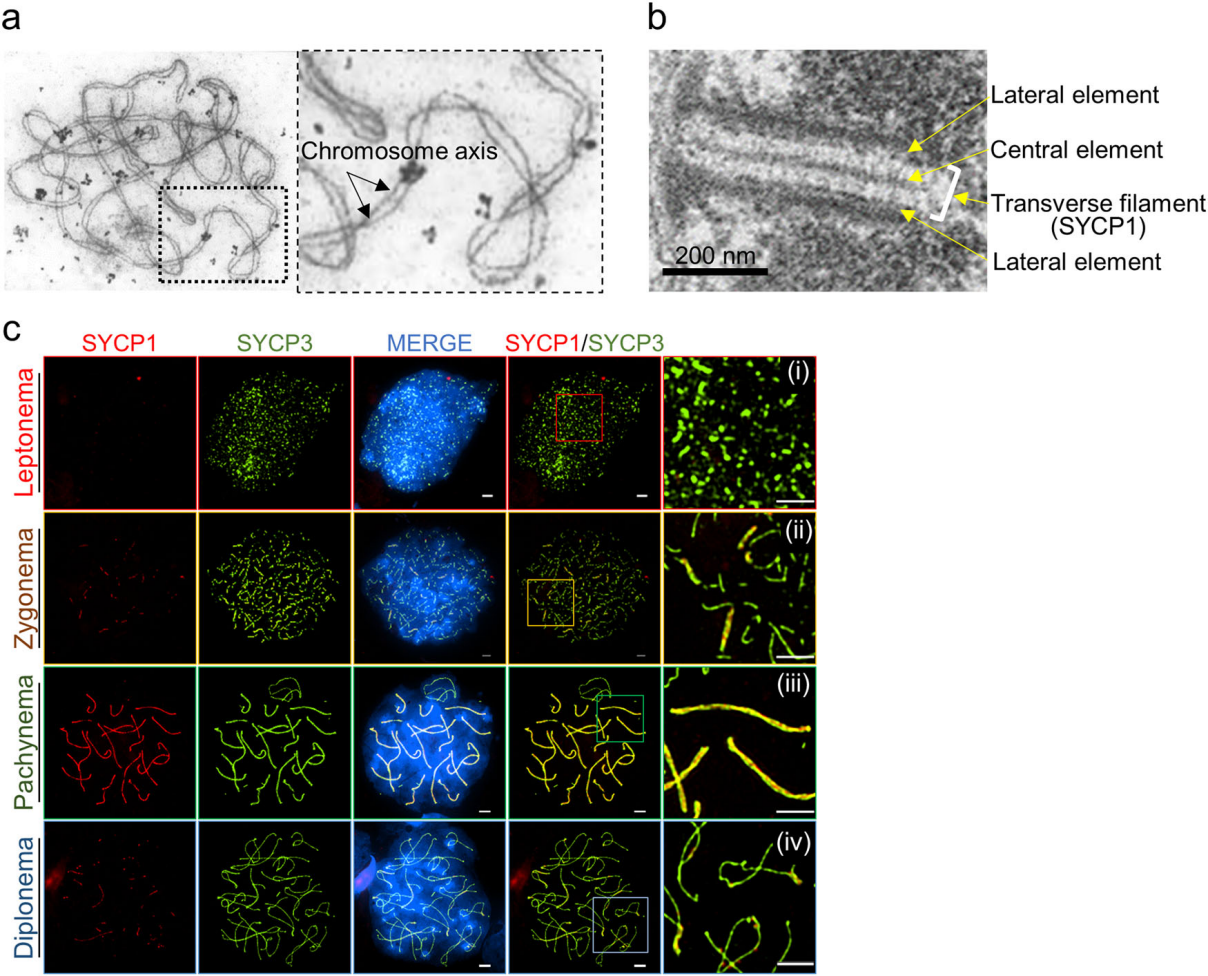
Chromosome organization during meiotic prophase I. **a** Structure of pachynema chromosomes in *Arabidopsis thaliana*. Chromosome axes at the pachynema stage were observed in electron micrographs^64^. **b** Ultrastructure of mouse SC consisting of transverse filaments with two lateral elements, separated by a central element (modified from Kouznetsova et al.^23^). Scale bar represents 200 nm. **c** Localization of SYCP1 and SYCP3 on chromosomes in meiotic prophase I. Spermatocytes of C57/BL6J mice were used for chromosome spreads. Cytological progression through meiotic prophase I was analyzed by immunostaining with anti-SYCP3 and anti-SYCP1 antibodies. (i–iv) Magnified views of overlay images from leptonema, zygonema, pachynema, and diplonema stages. SYCP3 was used to visualize chromosome axes for all stages of meiotic prophase I

**Fig. 2:**
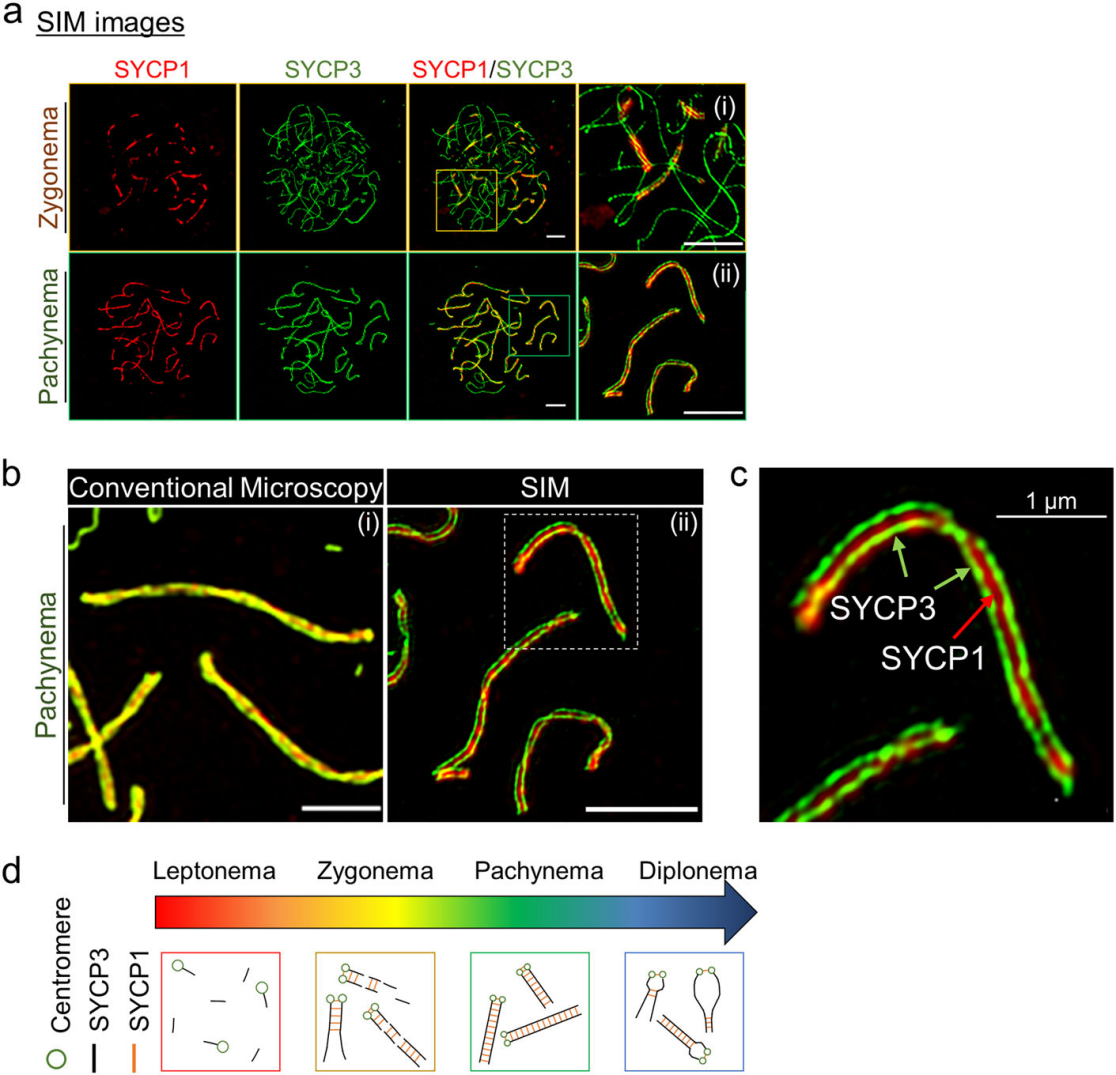
Super-resolution imaging of SYCP1 and SYCP3. **a** Immunofluorescence imaging of SCs by structured illumination microscopy (SIM). Magnification images of zygonema and pachynema are shown in (i) and (ii), respectively. Bars represent 2.5 μm. **b** Comparison of conventional microscopy and SIM for zygonema and pachynema. Panel (i) is the image from the conventional microscopy and panel (ii) is that acquired by SIM. Bars represent 2.5 μm. **c** Magnified images from **b** (ii) stained with anti-SYCP1 and anti-SYCP3 in pachynema. SYCP1 and SYCP3 were used to visualize central elements and chromosome axes for all stages of meiotic prophase I. **d** Schematic diagram of SC formation in meiotic prophase I. From the leptonema to the pachynema, the two lateral elements with a central element are assembled into SC with meiosis-specific proteins, including SYCP1 and SYCP3. In the diplonema stage, SC disassembly, induced by the dissociation of SC proteins, ensues and homologous chromosomes begin to separate, but they are held together by chiasmata

The structures of the chromosome axis in the pachynema of prophase I were determined by electron microscopy (EM) (Fig. 1a, b). EM analysis showed that the SC was assembled between homologous chromosomes comprising a tripartite structure of TFs (Fig. 1b)^23^. Multi-protein components including SYCP2/SYCP3, the cohesin complex, and HORMA (Hop1, Rev7, and Mad2) domain-containing protein 1 structurally stabilized the two LEs separated by a CE (Fig. 1a, b)^24–28^. To investigate chromosome morphogenesis during meiotic prophase I, conventional immunofluorescence microscopy was used to obtain images of the chromosome spreads. Nuclei were stained with antibodies against SYCP3 and SYCP1 on the chromosomes (Fig. 1c). Consistent with previous results, SYCP3 appeared during the leptonema stage and remained on the chromosome axis until diplonema (Fig. 1c)^27^. SYCP1, a CE component, was not observed during the leptonema stage (i) but appeared at the zygonema stage (ii). The full-length SC assembles completely at the pachynema (iii) and then disassembles at the diplonema (iv) (Fig. 1c). In general, imaging by conventional microscopy cannot produce precision images because of the diffracted fluorescence; staining of SYCP3 and SYCP1 revealed overlapped lines of the SC. We demonstrated that SYCP1 appeared between SYCP3 at the zygonema and axis elements were fully synapsed (Fig. 2). Recently, SIM has enabled fluorescence imaging of cellular structures at the nanometer scale. SIM imaging can generate images of SC formation that are considerably more detailed than those obtained via conventional microscopy (Fig. 2a–c). At the zygonema and pachynema stages, the two LEs were observed as separated lines in the EM images, and the CE stained with SYCP1 was localized in an internal border between LEs. Based on the SIM imaging results for SYCP3 and SYCP1 staining, we propose that structural changes occur for prophase chromosomes, and the suggested diagrams agree with the previous EM image data (Figs. 1b and 2). SYCP3 assembly begins at leptonema and continues through zygonema, during which SYCP1 begins to accumulate. By the pachynema stage, all chromosomes are fully synapsed (overlapped SYCP1-SYCP3 in conventional microscopy; separated SYCP1-SYCP3 in SIM). At the diplonema stage, the CEs are disassembled, while the chromosome LE remains attached at chiasmata sites (Fig. 2d). Thus, SIM imaging revealed the molecular organization of the SCs with nanometer precision for the meiotic chromosome.

### γH2AX occurs on asynapsed regions of chromosome axes

**Fig. 3:**
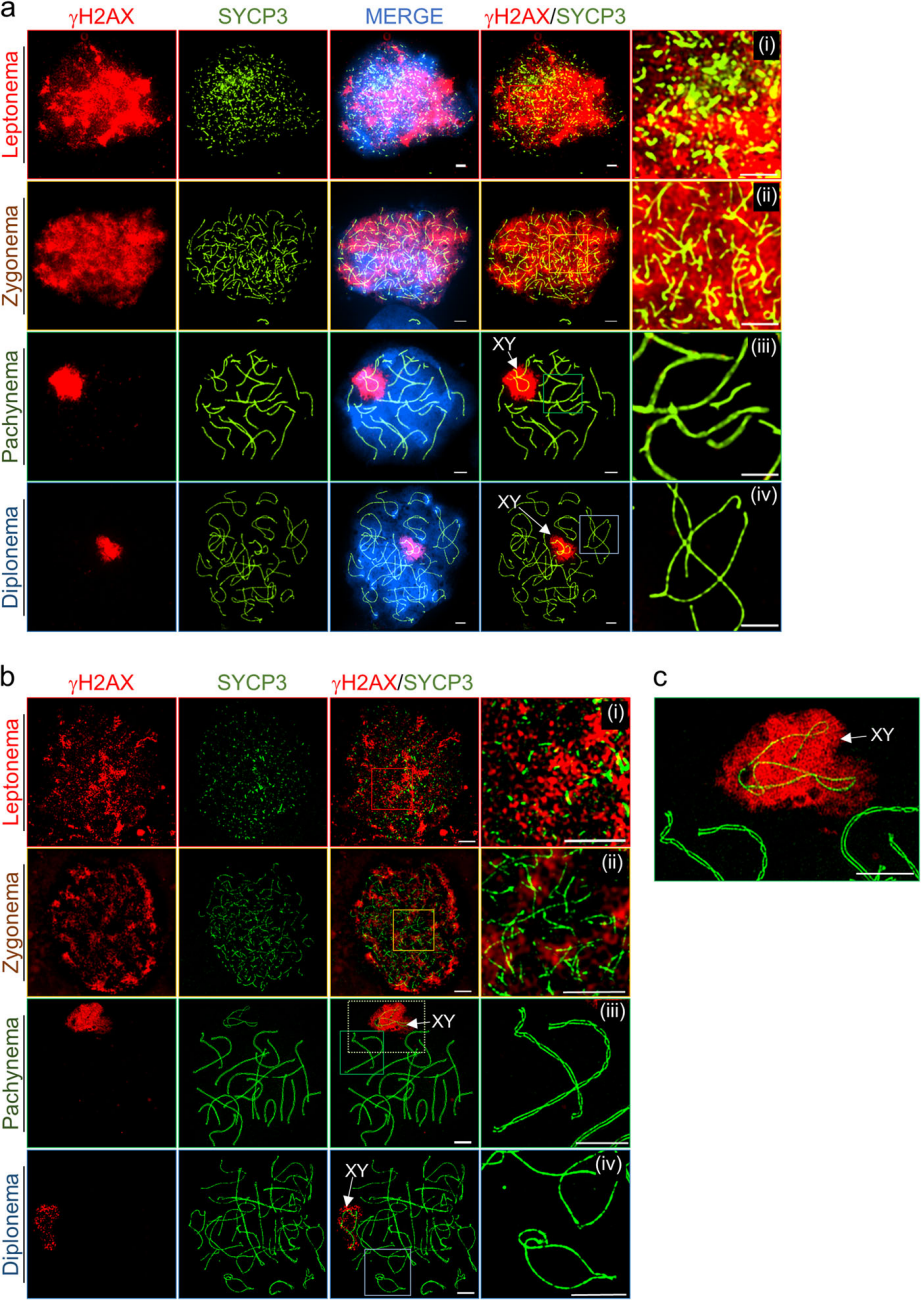
DSB repair and pairing of X–Y chromosomes occur later than those of autosomal chromosomes. Imaging analysis of distribution of SYCP3 and phosphorylated H2AX (γH2AX) in wild-type spermatocytes during prophase I of meiosis. Nuclei were simultaneously immunostained with anti-SYCP3 (green)/anti-γH2AX (red) and analyzed by conventional microscopy (**a**) and super-resolution microscopy (**b**) in prophase stages leptonema (i), zygonema (ii), pachynema (iii), and diplonema (iv). **c** Magnified image corresponding to colored box of pachynema chromosomes (**b**, iii). XY indicates male sex chromosomes. Bars represent 2.5 μm

**Fig. 4:**
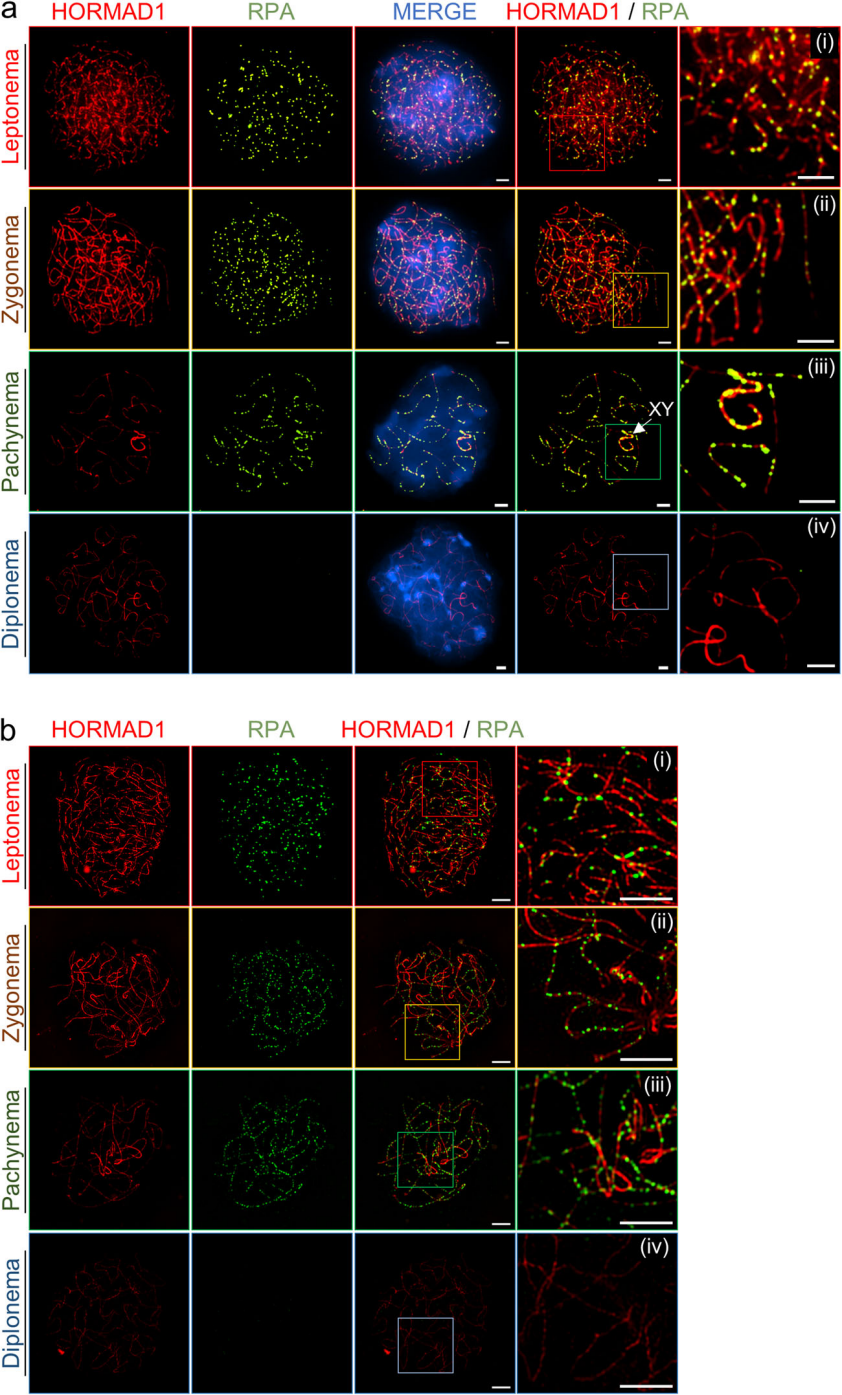
Distribution of replication protein A foci and HORMAD1 in prophase I. Imaging analysis of distribution of RPA and HORMAD1 in wild-type spermatocytes during prophase I of meiosis. Nuclei were simultaneously immunostained with anti-RPA (green)/anti-HORMAD1 (red) and analyzed by conventional microscopy (**a**) and super-resolution microscopy (**b**) in the prophase stages leptonema (i), zygonema (ii), pachynema (iii), and diplonema (iv). Bars represent 2.5 μm

Meiotic recombination is initiated by the formation of programmed DSBs catalyzed by Spo11^29,30^. Genome-wide mapping analysis has identified 15,000–20,000 DSB hotspots in the mouse genome^31,32^. Mouse spermatocytes generate 200–250 DSBs per cell^33,34^. During leptonema, H2AX, a member of the histone H2A histone family, is phosphorylated at serine 139 (known as γH2AX) by ataxia telangiectasia mutated (ATM) following Spo11-induced DSB formation^35–37^. Thus, γH2AX may be a sensitive target, indicating the response of DSBs on chromosomes, and DSB formation can be monitored via the temporal pattern of the appearance of γH2AX. To investigate DSB formation during prophase I, phosphorylated H2AX focus formation was observed by staining chromosome spreads with an anti-γH2AX antibody. During leptonema, programmed DSBs form and remain until the zygonema stage (Fig. 3a, b). Most γH2AX staining was lost at pachynema, whereas strong γH2AX signals remained in X–Y sex chromosomes, contained in a short segment known as the pseudoautosomal region (PAR) (Fig. 3)^38^. Given these results, DSBs of X–Y chromosomes disappear later than autosomal DSBs, indicating that PAR pairing occurs later than autosomal pairing (Fig. 3a). During the pachynema stage, synapsis is delayed for PARs, suggesting that X–Y chromosomes remain separate. During the diplonema stage, γH2AX remains in the X–Y chromosome but is completely dissociated in autosomes (Fig. 3a). SIM images also show a detailed similarity with conventional images (Fig. 3b, c). During the leptonema and zygonema stages, γH2AX signals are observed in most domains of the chromosomes. At the pachynema and diplonema stages, γH2AX is concentrated in the X–Y chromosome (indicated by the arrow), and many parts of X–Y chromosome axes are unpaired. HORMAD proteins, HORMAD1 and HORMAD2, are abundant meiotic proteins that localize to unsynapsed chromosome axes during meiotic prophase I^28^. Using conventional immunofluorescence microscopy, we observed that HORMAD1 was colocalized with SYCP3 during meiotic prophase I, that HORMAD1 preferentially accumulates on LEs at the zygonema or X–Y chromosome at the pachynema, and that HORMAD1 on chromosome axes were depleted by SC formation (Supplementary Figure 2). To determine the precise localization of RPA and the relationship between RPA and HORMADs during prophase I, we detected HORMAD1 and RPA by SIM imaging analysis (Fig. 4b). RPA colocalized with HORMAD1, specifically at the zygonema, and abundant RPA proteins localized to LEs, which strongly stained for HORMAD1 on meiotic chromatin (Fig. 4a, b). Interestingly, at the pachynema stage, most RPA foci appeared on the SC structure, while HORMAD1 proteins were depleted. Instead, HORMAD1 localized to unsynapsed regions of the X–Y sex chromosome. Thus, ssDNA or persistent recombination is not essential for the distribution of HORMAD1 along the chromosome axis. Based on the localization of HORMAD1 and RPA, this analysis reveals the chromosome structure and ssDNA dynamics.

### RPA focus formation in SC nucleation/polymerization

#### RPA localizes between LEs

**Fig. 5:**
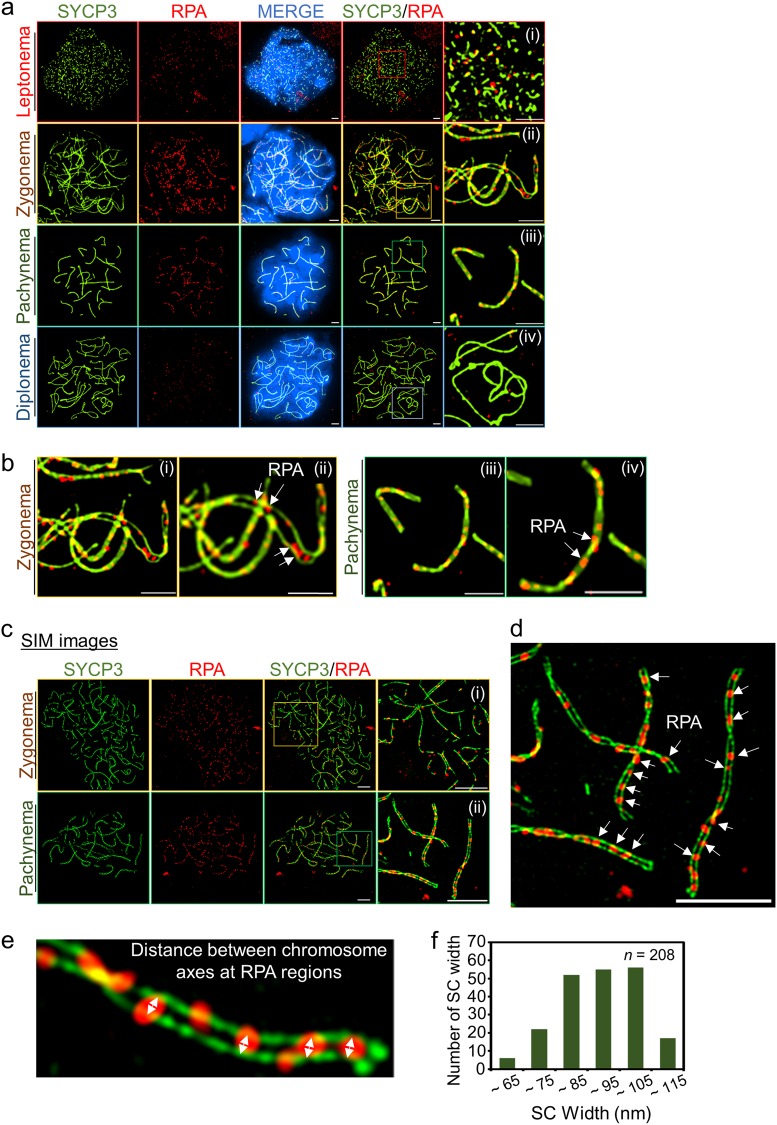
Distribution of replication protein A foci in prophase I. **a** RPA localization in leptonema (i), zygonema (ii), pachynema (iii), and diplonema (iv). The images were acquired via conventional fluorescence microscopy. Bars represent 2.5 μm. **b** Chromosome structures stained with anti-RPA and anti-SYCP3 antibodies. The images showing zygonema and pachynema chromosomes were magnified with overlay (**a**). White arrows indicate RPA foci. Bars represent 2.5 μm. **c** Super-resolution microscopy images of SYCP3 and RPA. Distribution of RPA on chromosomes was observed via SIM. Spermatocytes were stained with anti-RPA (red) and anti-SYCP3 (green) antibodies, and immunofluorescence images were acquired using SIM. (i) zygonema and (ii) pachynema. Bars represent 2.5 μm. **d** Overlay of magnified image at the pachynema stage. The image is enlarged from **c** (ii). White arrows represent RPA foci. Bars represent 2.5 μm. **e** Representative images showing chromosome axes and RPA foci. The arrows indicate the width of SCs at RPA-binding regions. **f** Quantification of distance between chromosome axes with RPA regions

RPA is involved in homologous recombination and acts with multiple proteins in the early phase of repair after a DSB is generated. In meiotic recombination, recombination nodules (RNs), multicomponent proteinaceous regions, are positioned at the CE of SC. These RNs can effectively be marked by RPA, Rad51, and Dmc1 and may be involved in homolog synapsis and meiotic recombination^38,39^. To investigate RPA localization in relation to RNs, we analyzed the dynamics of RPA focus formation (Fig. 5). Numerous RPA foci accumulated at the leptonema and began to load on the chromosome axes (confirmed by SYCP3 staining) starting at the zygonema (Fig. 5a, b). At the pachynema, most RPA proteins completely localized on the chromosome axes and were displaced during diplonema (Fig. 5a, b). However, RPA localization (whether they were on the axes or the CE of SC) could not be precisely determined by conventional microscopy. Thus, we obtained images of RPA localization using SIM (Fig. 5c–e). We predicted that RPA foci were located between LEs at the zygonema and pachynema stages of meiocytes. SIM imaging analysis showed that RPA proteins were located along each side of the single chromosome axis at zygonema, and RPA was between the two chromosome axes at pachynema, contributing to the assembly of homologous chromosomes (Fig. 5c, d).

#### RPA localizes to CE regions

Because LEs and CEs cannot be resolved as separated individual shapes in most cases of microscopy imaging, LEs and CEs were co-stained on a single structure. However, as we illustrated using EM imaging, the CEs localize to the inner center between LEs, as observed by anti-SYCP3 and anti-SYCP1 antibody staining (Fig. 2). To define the localization of RPA using super-resolution microscopy, we observed the immunofluorescence images using SIM for RPA and SYCP3. We found that RPA foci were localized to the inner side of the SYCP3 structure, indicating that the biochemical function of RPA occurs close to the CEs; this was consistent with immunogold EM imaging analysis^39^. Full SYCP3 assembly did not occur in the leptotene stage, but RPA foci were highly abundant because post-DSB formation requires additional end processing, including 5′ end resection and communication with modulator proteins (Fig. 5a–d). However, RPA localized on the chromosome axes stained with SYCP3, indicating that ssDNA ends of a DSB from a chromosome search for homologs and RPA in CEs began to appear in the zygonema (Fig. 5a). In the pachynema, most RPA foci localized to SYCP3-stained SC structures (Fig. 5a, b). In the last stage in prophase I, RPA foci were displaced from SYCP3 and were therefore not detectable on SCs (Fig. 5a, b). To elucidate the structural organization of the SCs and RPA localization with nanometer precision, we measured the width of the LE at the RPA region in wild-type meiocytes (Fig. 5e), revealing an average size of 93.24 ± 12.2 nm (*n* = 208) (Fig. 5f). These observations are consistent with previous EM analysis and indicate that RPA binding to CE regions is useful for monitoring meiotic recombination, particularly during the DSB-to-JM transition. It is important to note that the size of RPA foci varied from 173.65 to 273.97 nm in diameter, indicating variable lengths of ssDNA exposed on the status of recombination intermediates (Supplementary Figure 1).

#### RPA foci are dramatically reduced in pachynema but remain in SCs

**Fig. 6:**
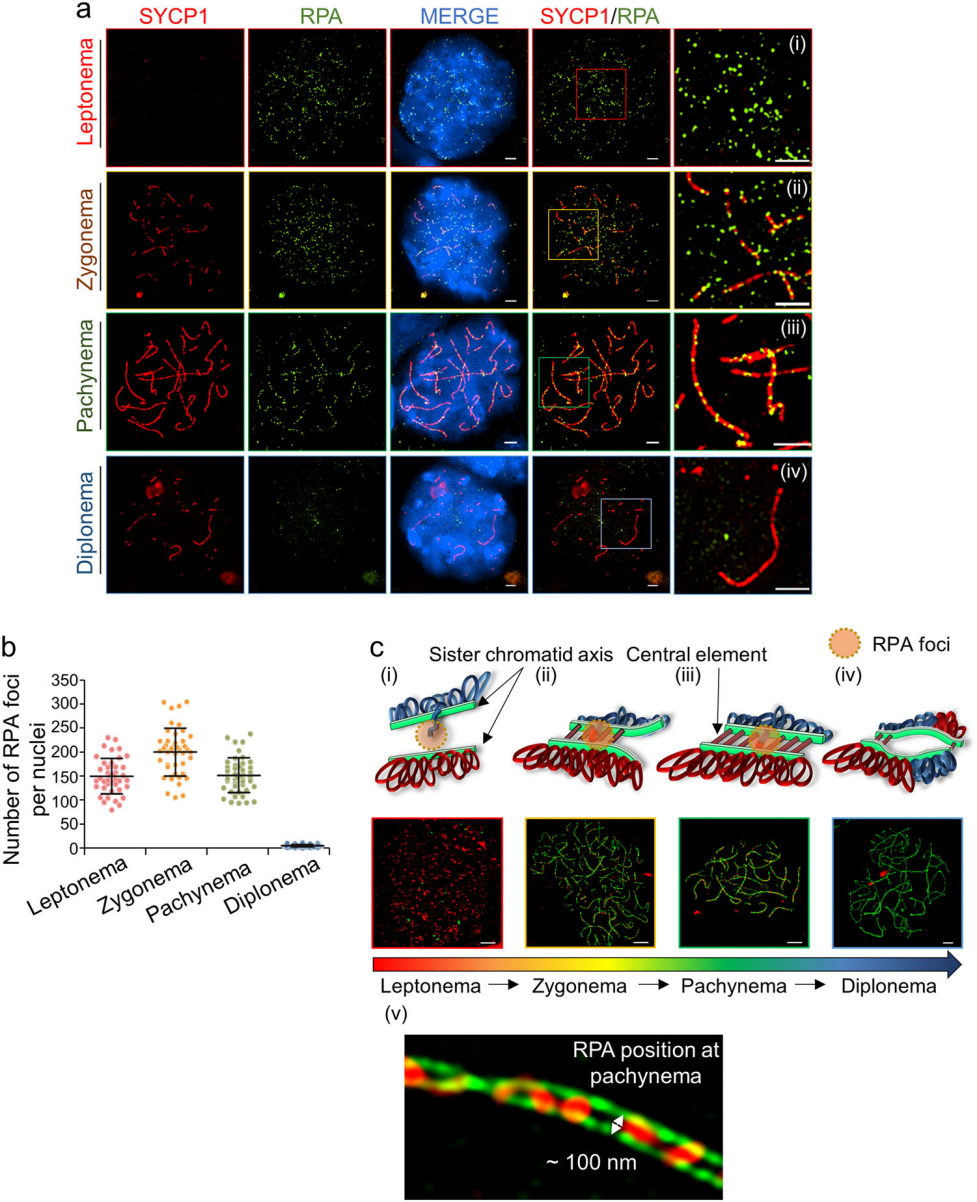
Change in the number of RPA foci from leptonema stage to diplonema stage. **a** Spermatocyte stained with antibodies against SYCP1 (red) and RPA (green). Nuclei were confirmed by conventional microscopy, and each box is magnified in (i)–(iv). (i) Leptonema stage, (ii) zygonema stage, (iii) pachynema stage, and (iv) diplonema stage. Bars represent 2.5 μm. **b** The temporal distribution of RPA in prophase I stage. The RPA foci were quantified and analyzed with the Prism 5 software. The scatter plot shows the number of RPA foci per nucleus. Error bars represent mean ± SD. **c** A model for the structural change of meiotic chromosomes and RPA localization in prophase I (i–iv). SIM images of RPA foci and SC width in prophase I (v). As shown by the electron microscopy images in Fig. 1b, the distance between lateral elements is approximately 100 nm in the SIM image

During meiosis, ssDNA is generated at multiple stages of recombination and is regulated by diverse factors that ensure its protection and subsequent steps of recombination. To examine the existence and properties of RPA foci on meiotic chromosomes, we analyzed the variation in the number of RPA foci at each stage of prophase I (Fig. 6a, b). Based on our quantification results, the number of RPA foci was 149.49 ± 37.30 during the leptonema stage, which increased to 199.91 ± 49.98 at the zygonema stage. Unexpectedly, we found that RPA foci (151.33 ± 36.56) remained on SC of the pachynema cells, supporting the presence of recombination intermediates or unrepaired DSBs at this stage. Most RPA loaded between chromosome axes was displaced at the diplonema stage, in contrast to in the leptonema-to-pachynema stages (Fig. 6a, b). Thus, most DSBs are likely correctly processed to recombinants (CO and/or non-crossover (NCO)). Assembly of RPA prevents ssDNA bonding to both DSB ends and the D-loop, and Rad51/Dmc1 require the displacement of RPA from nucleofilaments. In summary, we precisely monitored the dynamic changes in ssDNA and recombination progression. Our data suggest a model in which RPA localization occurs within the RN of SCs during prophase I and dynamically organizes the ssDNA of recombination intermediates (Fig. 6c).

## Discussion

Meiosis is a reduction division that is essential for genetic diversity and sexual reproduction. Meiotic prophase I is an important stage for recombination and proper chromosome segregation, and abnormal progression in this stage causes genetic diseases and chromosomal abnormalities known as genomic disorders. Chromosomal events and meiotic recombination are coordinated to ensure various processes of homolog pairing, synapsis, DSB repair, and chromosome segregation during meiotic prophase I to form the SCs between homologous chromosomes and mediate chromosomal and recombinational processes. Thus, high-resolution cytological analysis is required to understand the relationship between the SC structure and RN, which contains diverse recombination-associated proteins in meiotic prophase I. However, SC substructures, LEs and CEs, and the dynamics of recombination progression cannot be resolved at the nanometer scale by conventional microscopy. In this study, using SIM imaging, we determined the organization of SCs and their interactions with RPA in recombination during meiosis.

Meiotic events may involve combining the DNA events of DSB repair and the chromosome structural changes of prophase with recombination that targets changes to specific sites, while the robust meiosis-specific chromosome structure inhibits the occurrence of these dramatic changes outside of meiosis. SC nucleation occurs preferentially at sites of CO designation. Previous studies using conventional microscopy revealed RPA localization on SCs in meiosis^23,39–41^. Here, we determined the detailed RPA localization together with the dynamics of SC by SIM. More than 200 RPA foci that were programmed DSB regions appeared at the leptonema/zygonema stage and firmly localized on CEs of SC at the pachynema, indicating RPA localization at the recombinosome complex (or RN). Furthermore, LEs stained with SYCP3 were observed as a line; however, the axis proteins had two lines juxtaposed with each line at the pachytene stage because SYCP3 is essential for the formation of chromosome axes and SC nucleation^16,42^. Generally, it is difficult to observe juxtaposed SYCP3 at the pachytene stage by conventional microscopy. We obtained SIM images showing that two single chromosome axes and RPA specifically localized between the LEs of SC structures, and we determined the size and the subcellular distrubution of RPA in prophase I of meiocytes at precision resolution.

Assembly and disassembly of SC occur in meiotic prophase I, and meiotic recombination is modulated by SC dynamics. Spo11 catalyzes the formation of programmed DSB at the leptonema stage, in response to which ATM phosphorylates a histone component, H2AX. Therefore, the appearance of γH2AX indicates the genomic regions of DSBs. Most meiotic DSBs are repaired via the homologous recombination pathway, and chromosome morphology must be changed to support recombination and homolog pairing^2,43–46^. During the leptonema-to-zygonema transition, recombination-mediated DSB repair is initiated, and recombination proteins, including RPA, Rad51, and Dmc1, are loaded on RNs. These proteins play important roles in meiotic recombination, specifically during homolog search and synapsis^7,47–49^. Because RPA firmly binds to ssDNAs and inhibits the formation of DNA secondary structures^50,51^, its dynamics can be used to monitor recombination progression^23,52,53^. Based on these dynamics, ssDNAs are generated by programmed DSBs, and RPA localizes CEs in between chromosome axes during the pachynema, gradually reducing the number of RPA foci during the repair process. This is because DSBs pair with the homolog template and because the joint molecules and second DSB ends are properly processed; thus, most RPA proteins are displaced from SCs at the diplonema, indicating that meiotic recombination is complete.

Several studies have revealed the correlation between SYCP1 and SYCP3 and that multiple proteins are required for SC formation during prophase I of meiocytes. Diverse TF proteins have been reported SYCP1 in mice; Zip1 in budding yeast; SYP-1, SYP-2, SYP-3, and SYP-4 in *Caenorhabditis elegans*; ZYP1a and ZYP1b in *Arabidopsis thaliana;* C(3)G in *Drosophila melanogaster*^54–57^. As stated above, SYCP3 is a chromosome axis protein that localizes along the SC; SYCP2 also localizes in the axes^42,58–60^. In mice, SYCP1 is a major TF that connects homologous chromosomes (“pairing”)^8,9^. At the leptotene stage, SC begins assembling: SYCP3 localizes along each chromosome axis and SYCP1 can interact with the chromosome axis to which diverse proteins are localized including SYCP2, SYCP3, cohesin complex, and HORMAD proteins in the zygotene stage^8,24,42,61^. SC is dynamically assembled during the homolog pairing process and disassembled at the end of prophase I after CO/NCO appears, suggesting that SYCP1 is the core factor for polymerization of SC and recombination.

Sex chromosomes exhibit a different phenomenon that delays pairing and prolongs the appearance of γH2AX. In X–Y chromosomes, γH2AX remains until not only the pachynema, but also the diplonema stage. In contrast, in autosomes, most γH2AX foci disappear at the pachynema and diplonema stages. This suggests that meiotic recombination is delayed and occurs differently in sex chromosomes vs. autosomal chromosome. Sex chromosomes have regions known as PAR, which are a large part of the X chromosome and Y chromosome that do not contain homologous regions, produced during genetic recombination^62,63^. Thus, they are unable to pair completely and DSBs remain until the end of prophase I in sex chromosomes.

The present results suggest that the dynamics of RPA localization are modulated in the chromosome axes at the early leptonema/zygonema stages; full SCs, an important feature of CO-designated recombination in meiotic recombination, form at the mid-pachynema. The precise resolution of RPA localization as a marker of ssDNA in meiotic prophase I has not been determined because conventional microscopy imaging techniques were limited in resolving their molecular specificity. Using SIM imaging, we determined the detailed structure of SC and alterations in RPA localization at the nanometer scale. Further studies are needed to examine the cellular dynamics of recombinase and chromosome structural components in meiotic recombination and understand chromosome morphogenesis in the homolog searching and pairing processes.

## Electronic supplementary material

Supplemental Information
