# Supplementary material for: Structured illumination microscopy imaging reveals localization of replication protein A between chromosome lateral elements during mammalian meiosis
Source: Exp Mol Med. 2018 Aug 28;50(8):112. doi: 10.1038/s12276-018-0139-5 (PMC6113238; doi:10.1038/s12276-018-0139-5)
Supplement: Supplementary file 1 — Supplemental Information [file 12276_2018_139_MOESM1_ESM.docx]

**Supplemental Information**

**Structured Illumination Microscopy Imaging Reveals Localization of Replication Protein A between Chromosome Lateral Elements during Mammalian Meiosis**

Seobin Yoon^1^, Eui-Hwan Choi^1^, Jung-Woong Kim^1^, and Keun P. Kim^1,*^

^1^Department of Life Sciences, Chung-Ang University, Seoul 06974, Korea

*To whom correspondence should be addressed.


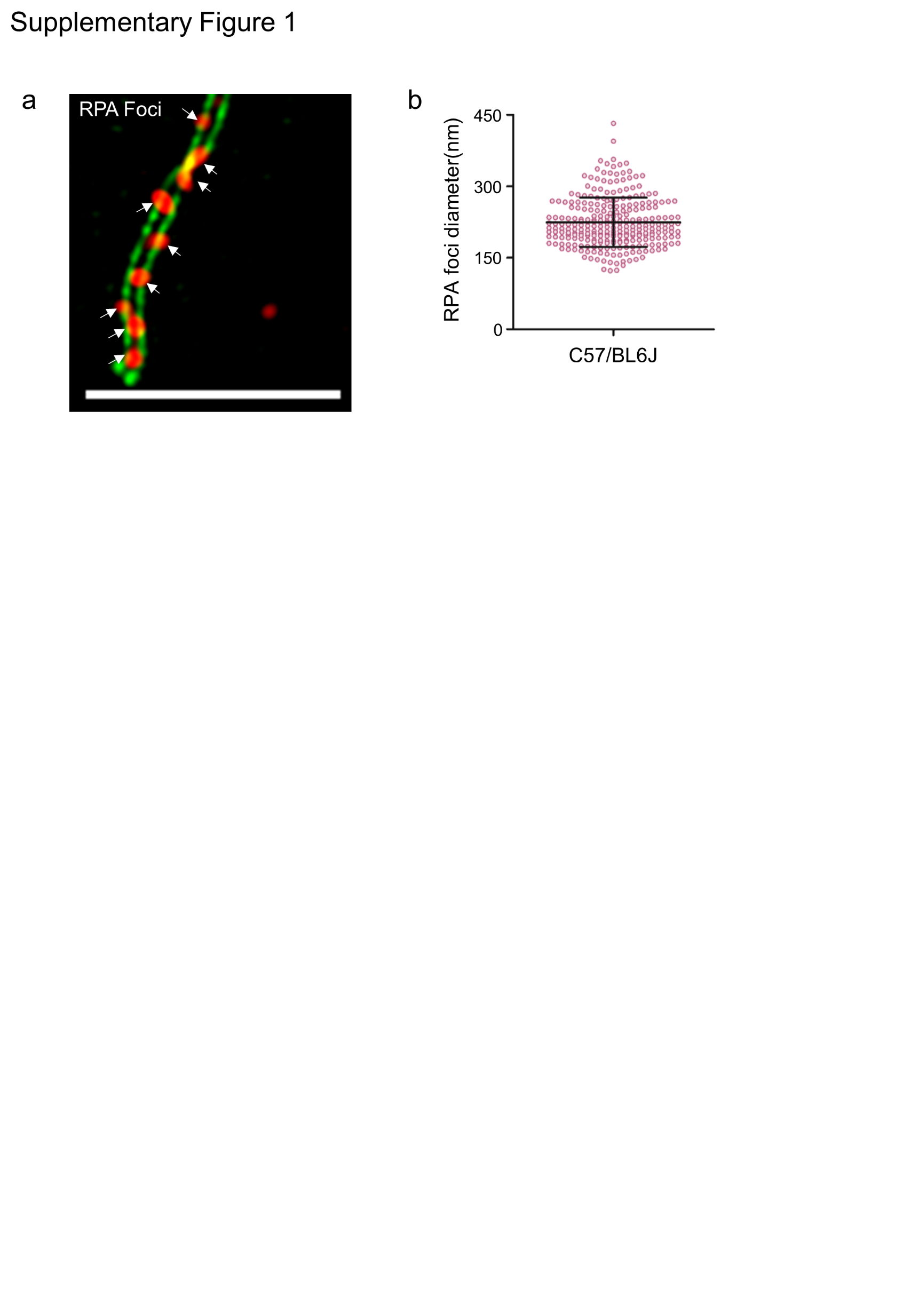


**Supplementary Figure 1. Diameter of RPA foci in pachynema.**

(a) Representative image of pachynema stained with anti-RPA antibody. Bars represent 2.5 μm.

(b) Size distribution of RPA foci at pachynema stage. Spermatocyte was stained with anti-RPA antibody and the size of RPA foci was analyzed, using Nikon NIS software. The scatter plot shows the distribution of RPA foci in size (n = 274).


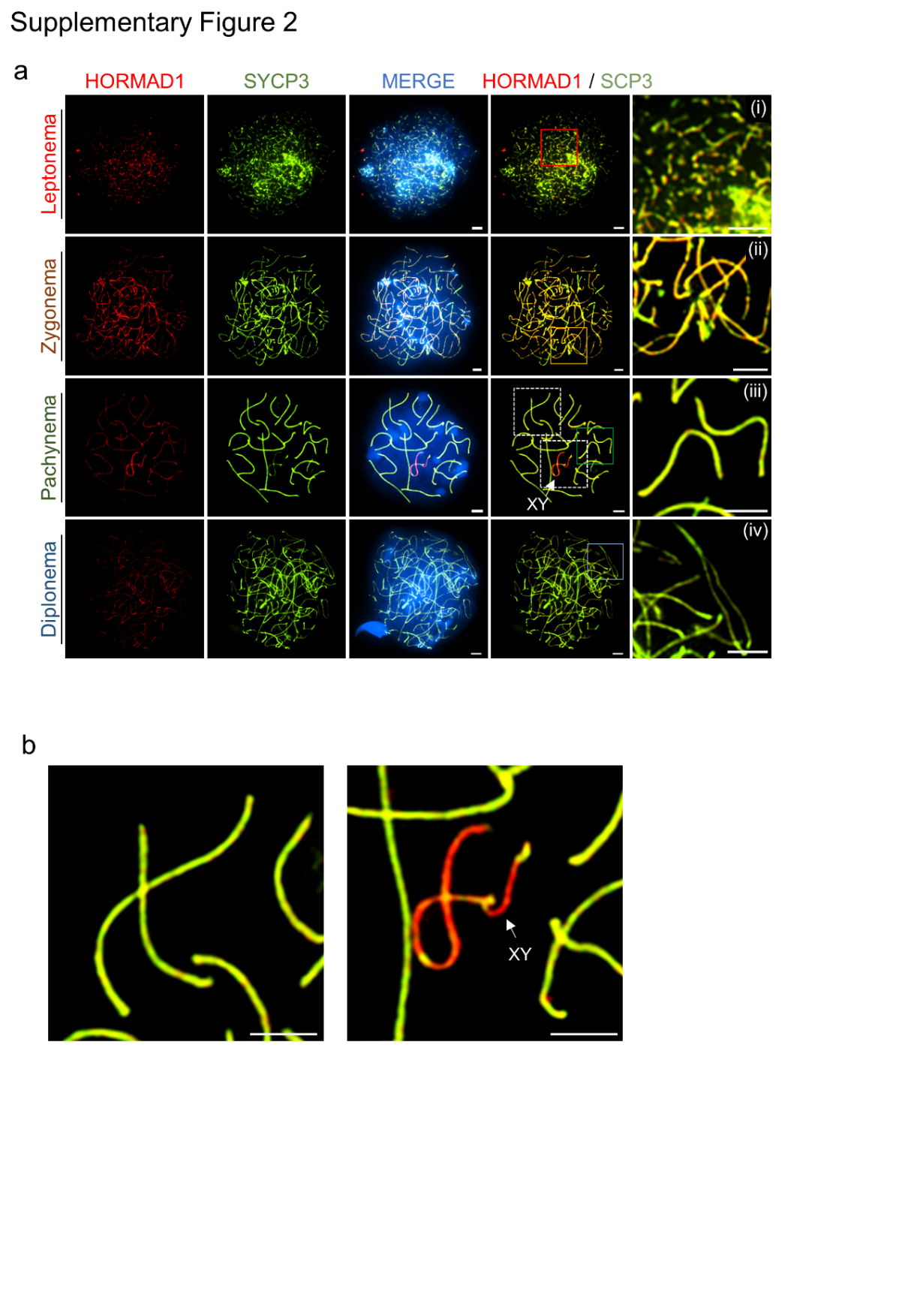


**Supplementary Figure 2. Localization of HORMAD1 and SYCP3 on chromosome axes.**

(a) Localization of HORMAD1 and SYCP3 on chromosomes in meiotic prophase I. Spermatocytes of C57/BL6J mice was used for chromosome spreads. Cytological progression through meiotic prophase I were analyzed by immunostaining with anti-SYCP3 and anti-SYCP1 antibodies. (i–iv) Magnification views of overlay images from leptonema, zygonema, pachynema and diplonema stages. SYCP3 was used to visualize chromosome axes for all stages of the meiotic prophase I. White arrow represents X-Y chromosomes. Bars represent 2.5 μm.

(b) Magnification images of pachynema are shown in (iii). White arrow represents X-Y chromosomes. Bars represent 2.5 μm.
